# Crystal structure and Hirshfeld surface analysis of hexyl 1-hexyl-2-oxo-1,2-di­hydro­quinoline-4-carboxyl­ate

**DOI:** 10.1107/S2056989020004521

**Published:** 2020-04-09

**Authors:** Younos Bouzian, Sevgi Kansiz, Lhassane Mahi, Noureddine Hamou Ahabchane, Joel T. Mague, Necmi Dege, Khalid Karrouchi, El Mokhtar Essassi

**Affiliations:** a Laboratory of Heterocyclic Organic Chemistry URAC 21, Pole of Competence Pharmacochemistry, Av Ibn Battouta, BP 1014, Faculty of Sciences, Mohammed V University, Rabat, Morocco; b Department of Fundamental Sciences, Faculty of Engineering, Samsun University, Samsun 55420, Turkey; c Moroccan Foundation for Advanced Science Innovation and Research (Mascir), Department of Nanotechnology, Rabat Design Center, Rue Mohamed Al Jazouli-Madinat Al Irfane, Rabat 10 100, Morocco; d Department of Chemistry, Tulane University, New Orleans, LA 70118, USA; e Department of Physics, Faculty of Arts and Sciences, Ondokuz Mayıs University, Samsun, 55200, Turkey; f Laboratory of Analytical Chemistry and Bromatology, Faculty of Medicine and Pharmacy, Mohamed V University, Rabat, Morocco

**Keywords:** crystal structure, di­hydro­quinoline, aliphatic chains, π-stacking, Hirshfeld surface analysis

## Abstract

The asymmetric unit of the title compound, C_22_H_31_NO_3_, comprises of one mol­ecule. The mol­ecule is not planar, with the carboxyl­ate ester group inclined by 33.47 (4)° to the heterocyclic ring. Individual mol­ecules are linked by _aromatic_C—H⋯O_carbon­yl_ hydrogen bonds into chains running parallel to [001]. Slipped π–π stacking inter­actions between quinoline moieties link these chains into layers extending parallel to (100). Hirshfeld surface analysis, two-dimensional fingerprint plots and mol­ecular electrostatic potential surfaces were used to qu­antify the inter­molecular inter­actions present in the crystal, indicating that the most important contributions for the crystal packing are from H⋯H (72%), O⋯H/H⋯O (14.5%) and C⋯H/H⋯C (5.6%) inter­actions.

## Abstract 2

The di­hydro­quinoline unit is slightly twisted and the hexyl groups extend out on either side. In the crystal, C—H⋯O hydrogen bonds form chains of mol­ecules extending along the *b*-axis direction, which are paired up by slipped π-stacking inter­actions. The ends of the hexyl groups from neighbouring chains are in contact but do not inter­calate.

## Chemical context   

Quinoline derivatives represent an important class of heterocyclic compounds utilized as pharmaceuticals (Chu *et al.*, 2019 ▸). They possess various biological properties such as anti­bacterial (Panda *et al.*, 2015 ▸), anti­cancer (Tang *et al.*, 2018 ▸), anti­tubercular (Xu *et al.*, 2017 ▸), anti­viral (Sekgota *et al.*, 2017 ▸), anti-HCV (Cannalire *et al.*, 2016 ▸), anti­malarial (Hu *et al.*, 2017 ▸), anti-Alzheimer’s (Bolognesi *et al.*, 2007 ▸), anti­leishmanial (Palit *et al.*, 2009 ▸) and anti-inflammatory (Pinz *et al.*, 2016 ▸) activities.
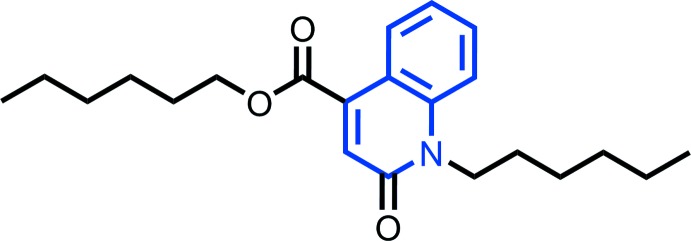


In view of the biological importance of quinoline, and in a continuation of our research work devoted to the syntheses and crystal structures of quinoline derivatives (Bouzian *et al.*, 2019**a* ▸,b* ▸), we report herein on the mol­ecular and crystal structures of hexyl 1-hexyl-2-oxo-1,2-di­hydro­quinoline-4-carb­oxyl­ate, (I), which was prepared by reacting ethyl 6-chloro-2-oxo-1,2-di­hydro­quinoline-4-carboxyl­ate with 1-bromo­hexane in the presence of a catalytic qu­antity of tetra-*n*-butyl­ammonium bromide. Inter­molecular inter­actions were qu­anti­fied by Hirshfeld surface analysis.

## Structural commentary   

**Figure 1:**
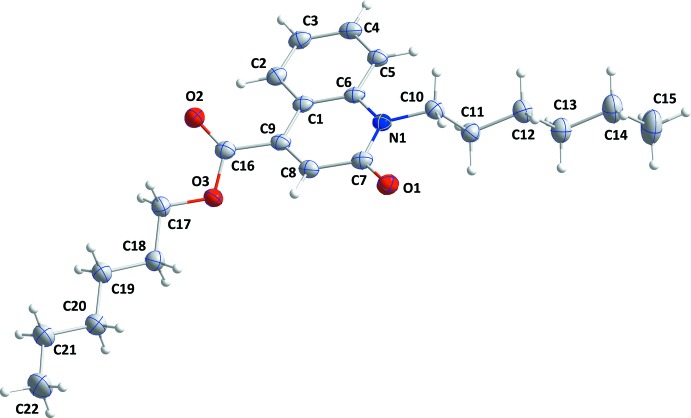
The title mol­ecule with displacement ellipsoids drawn at the 50% probability level.

The mol­ecule of (I) is shown in Fig. 1 ▸. It is non-planar, with the carboxyl ester group inclined by 33.47 (4)° to the heterocyclic ring (r.m.s. deviation of the ten atoms = 0.0174 Å). The hexyl chain attached to N1 is twisted out of this plane by 14.2 (2)° whereas the hexyl chain attached to O1 is twisted by 23.1 (2)° from this plane.

## Supra­molecular features   

**Figure 2:**
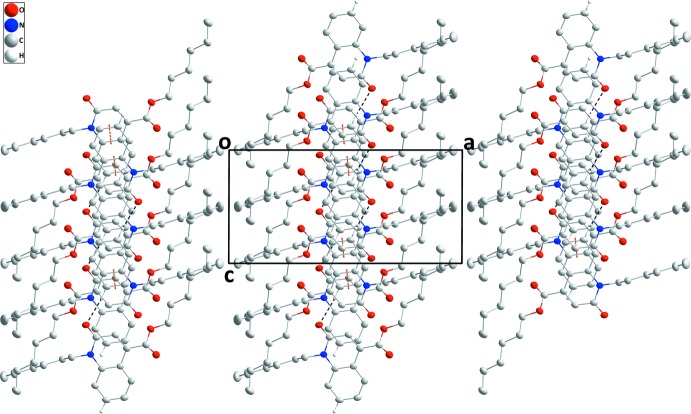
The crystal packing viewed along [010], with C—H⋯O hydrogen bonds and π–π stacking inter­actions indicated by black and orange dashed lines, respectively.

**Figure 3:**
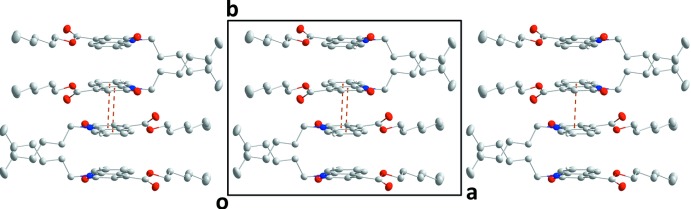
The crystal packing viewed along [001], with π–π stacking inter­actions indicated by orange dashed lines.

In the crystal, C4—H4⋯O1 hydrogen bonds between the phenyl ring and the carbonyl group of an adjacent mol­ecule lead to the formation of chains running parallel to [001] (Table 1 ▸, Fig. 2 ▸). These chains are connected in pairs along [010] through slipped π–π stacking inter­actions between inversion-related di­hydro­quinoline moieties [*Cg*1⋯*Cg*2^i^ = 3.5472 (9) Å with a slippage of 0.957 Å; *Cg*1 and *Cg*2 are the centroids of the N1/C6/C1/C9/C8/C7 and C1–C6 rings; symmetry code: (i) 1 − *x*, −*y*, 1 − *z*] (Figs. 2 ▸, 3 ▸). This way, (100) layers with a width corresponding to the length of the *a* axis are formed. Unlike the packing features of similar mol­ecules, the hexyl chains are not oriented in parallel. This is possibly a consequence of the π–π stacking inter­actions, which result in a ‘crossed’ orientation of neighbouring hexyl groups (Fig. 3 ▸).

## Database survey   

A search of the Cambridge Structural Database (CSD, version 5.40, update of August 2019; Groom *et al.*, 2016 ▸) using 2-oxo-1,2-di­hydro­quinoline-4-carb­oxy­lic acid as the main skeleton revealed five structures similar to the title compound. They contain the oxo­quinoline moiety with different substit­uents, *viz*. 2-oxo-1,2-di­hydro­quinoline-4-carb­oxy­lic acid monohydrate (EQAVAV; Filali Baba *et al.*, 2016 ▸), ethyl 1*H*-3-hy­droxy-2-oxo-1,2-di­hydro­quinoline-4-carboxyl­ate (RAV­JAA01; Paterna *et al.*, 2013 ▸), ethyl 1-methyl-2-oxo-1,2-di­hydro­quinoline-4-carboxyl­ate (SECCAH; Filali Baba *et al.*, 2017*a* ▸), prop-2-yn-1-yl 2-oxo-1-(prop-2-yn-1-yl)-1,2-di­hydro­quinoline-4-carboxyl­ate (XILYUP; Filali Baba *et al.*, 2017*b* ▸) and ethyl 1-benzyl-3-hy­droxy-2-oxo-1,2-di­hydro­quinoline-4-carboxyl­ate (ZINHEL; Paterna *et al.*, 2013 ▸). The layers present in EQAVAV are linked together by pairwise N—H⋯O inter­actions. In SECCAH, weak C—H⋯O hydrogen bonds link the mol­ecules into zigzag chains along [100]. A single weak C—H⋯O inter­molecular inter­action links the mol­ecules into [001] chains in XILYUP.

## Hirshfeld surface analysis   

**Figure 4:**
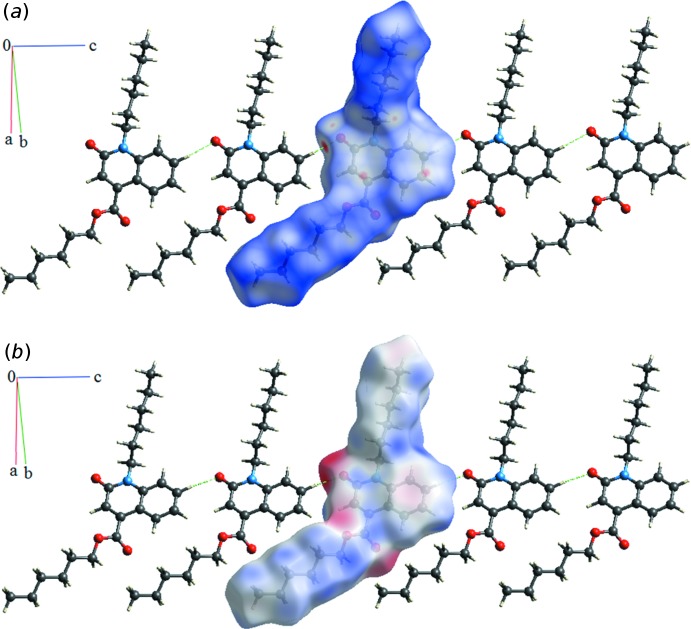
(*a*) The Hirshfeld surfaces of the title compound mapped over *d*_norm_, with a fixed colour scale of −0.1822 (red) to 1.3083 (blue) a.u., and (*b*) the Hirshfeld surface mapped over mol­ecular electrostatic potential showing C—H⋯O hydrogen bonds, with a fixed colour scale of −0.0733 (red) to 0.0381(blue) a.u..

**Figure 5:**
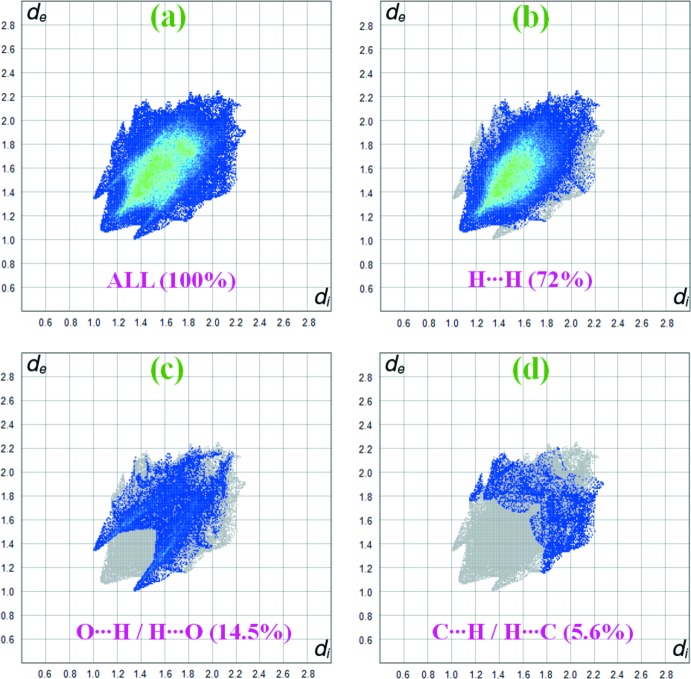
Two-dimensional fingerprint plots to the Hirshfeld surface with (*a*) a *d*_norm_ view for (I) and delineated into relative contributions for (*b*) H⋯H, (*c*) O⋯H/H⋯H and (*d*) C⋯H/H⋯C inter­actions.

To investigate the inter­molecular inter­actions, Hirshfeld surface analysis (Spackman & Jayatilaka, 2009 ▸) and two-dimensional fingerprint plots were generated for the mol­ecule using *CrystalExplorer17.5* (Turner *et al.*, 2017 ▸). Hirshfeld surface analysis depicts inter­molecular inter­actions by different colours, representing short or long contacts and further the relative strength of the inter­action. The generated Hirshfeld surface mapped over *d_norm_* is shown in Fig. 4 ▸ *a*. A view of the three-dimensional Hirshfeld surface of the title compound plotted over electrostatic potential, highlighting the C—H⋯O contacts, is given in Fig. 4 ▸ *b*. As revealed by the two-dimensional fingerprint plots (Fig. 5 ▸), the crystal packing is dominated by H⋯H contacts, representing van der Waals inter­actions (72% contribution to the overall surface), followed by O⋯H and C⋯H inter­actions, which contribute with 14.5% and 5.6%, respectively. The contributions of the C⋯C (5.4%), C⋯O (0.8%), C⋯N (0.7%) and N⋯H (0.6%) inter­actions are less significant.

## Synthesis and crystallization   

A mixture of 2-oxo-1,2-di­hydro­quinoline-4-carb­oxy­lic acid (0.5 g, 2.6 mmol), K_2_CO_3_ (0.73 g, 5.29 mmol), 1-bromo­hexane (0.66 g, 4 mmol) and tetra-*n*-butyl­ammonium bromide as catalyst in DMF (25 ml) was stirred at room temperature for 48 h. The solution was filtered by suction, and the solvent was removed under reduced pressure. The residue was chromatographed on a silica-gel column using hexane and ethyl acetate (*v*/*v*, 95/5) as eluents to afford (I). Single crystals were obtained by slow evaporation of an ethano­lic solution.

## Refinement   

Crystal data, data collection and structure refinement details are summarized in Table 2 ▸. All H atoms were located in difference-Fourier maps and were refined freely. 

## Supplementary Material

Crystal structure: contains datablock(s) global, I. DOI: 10.1107/S2056989020004521/wm5550sup1.cif

Structure factors: contains datablock(s) I. DOI: 10.1107/S2056989020004521/wm5550Isup2.hkl

CCDC reference: 1994187

Additional supporting information:  crystallographic information; 3D view; checkCIF report

## Figures and Tables

**Table 1:** Hydrogen-bond geometry (Å, °)

*D*—H⋯*A*	*D*—H	H⋯*A*	*D*⋯*A*	*D*—H⋯*A*
C4—H4⋯O1^i^	0.960 (17)	2.475 (17)	3.3670 (19)	154.7 (15)

Symmetry code: (i) 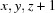.

**Table 2:** Experimental details

Crystal data	
Chemical formula	C_22_H_31_NO_3_
*M*_r_	357.48
Crystal system, space group	Monoclinic, *P*2_1_/*c*
Temperature (K)	150
*a*, *b*, *c* (Å)	17.6928 (7), 13.2512 (5), 8.5916 (3)
β (°)	90.184 (2)
*V* (Å^3^)	2014.30 (13)
*Z*	4
Radiation type	Cu *K*α
μ (mm^−1^)	0.61
Crystal size (mm)	0.25 × 0.17 × 0.10

Data collection	
Diffractometer	Bruker D8 VENTURE PHOTON 100 CMOS
Absorption correction	Multi-scan (*SADABS*; Krause *et al.*, 2015 ▸)
*T*_min_, *T*_max_	0.82, 0.94
No. of measured, independent and observed [*I* > 2σ(*I*)] reflections	14697, 3924, 3044
*R*_int_	0.048
(sin θ/λ)_max_ (Å^−1^)	0.618

Refinement	
*R*[*F* ^2^ > 2σ(*F* ^2^)], *wR*(*F* ^2^), *S*	0.046, 0.105, 1.07
No. of reflections	3924
No. of parameters	359
H-atom treatment	All H-atom parameters refined
Δρ_max_, Δρ_min_ (e Å^−3^)	0.19, −0.18

Computer programs: *APEX3* and *SAINT* (Bruker, 2016 ▸), *SHELXT/5* (Sheldrick, 2015*a* ▸), *SHELXL2018/3* (Sheldrick, 2015*b* ▸), *DIAMOND* (Brandenburg & Putz, 2012 ▸), *SHELXTL* (Sheldrick, 2008 ▸) and *publCIF* (Westrip, 2010 ▸).

## supplementary crystallographic information

### Crystal data

**Table:**

C_22_H_31_NO_3_	*F*(000) = 776
*M**_r_* = 357.48	*D*_x_ = 1.179 Mg m^−^^3^
Monoclinic, *P*2_1_/*c*	Cu *K*α radiation, λ = 1.54178 Å
*a* = 17.6928 (7) Å	Cell parameters from 9350 reflections
*b* = 13.2512 (5) Å	θ = 5.0–72.3°
*c* = 8.5916 (3) Å	µ = 0.61 mm^−^^1^
β = 90.184 (2)°	*T* = 150 K
*V* = 2014.30 (13) Å^3^	Block, colourless
*Z* = 4	0.25 × 0.17 × 0.10 mm

### Data collection

**Table:**

Bruker D8 VENTURE PHOTON 100 CMOS diffractometer	3924 independent reflections
Radiation source: INCOATEC IµS micro–focus source	3044 reflections with *I* > 2σ(*I*)
Mirror monochromator	*R*_int_ = 0.048
Detector resolution: 10.4167 pixels mm^-1^	θ_max_ = 72.4°, θ_min_ = 4.2°
ω scans	*h* = −21→21
Absorption correction: multi-scan (*SADABS*; Krause *et al.*, 2015)	*k* = −16→15
*T*_min_ = 0.82, *T*_max_ = 0.94	*l* = −9→10
14697 measured reflections	

### Refinement

**Table:**

Refinement on *F*^2^	Primary atom site location: dual
Least-squares matrix: full	Secondary atom site location: difference Fourier map
*R*[*F*^2^ > 2σ(*F*^2^)] = 0.046	Hydrogen site location: difference Fourier map
*wR*(*F*^2^) = 0.105	All H-atom parameters refined
*S* = 1.07	*w* = 1/[σ^2^(*F*_o_^2^) + (0.028*P*)^2^ + 0.8763*P*] where *P* = (*F*_o_^2^ + 2*F*_c_^2^)/3
3924 reflections	(Δ/σ)_max_ < 0.001
359 parameters	Δρ_max_ = 0.19 e Å^−^^3^
0 restraints	Δρ_min_ = −0.18 e Å^−^^3^

### Special details

**Table:**

Geometry. All esds (except the esd in the dihedral angle between two l.s. planes) are estimated using the full covariance matrix. The cell esds are taken into account individually in the estimation of esds in distances, angles and torsion angles; correlations between esds in cell parameters are only used when they are defined by crystal symmetry. An approximate (isotropic) treatment of cell esds is used for estimating esds involving l.s. planes.
Refinement. Refinement of F^2^ against ALL reflections. The weighted R-factor wR and goodness of fit S are based on F^2^, conventional R-factors R are based on F, with F set to zero for negative F^2^. The threshold expression of F^2^ > 2sigma(F^2^) is used only for calculating R-factors(gt) etc. and is not relevant to the choice of reflections for refinement. R-factors based on F^2^ are statistically about twice as large as those based on F, and R- factors based on ALL data will be even larger.

### Fractional atomic coordinates and isotropic or equivalent isotropic displacement parameters (Å^2^)

**Table:**

	*x*	*y*	*z*	*U*_iso_*/*U*_eq_	
O1	0.38830 (7)	0.40574 (9)	0.54321 (12)	0.0338 (3)	
O2	0.68998 (7)	0.45106 (10)	0.80686 (14)	0.0384 (3)	
O3	0.66596 (7)	0.37452 (8)	0.57906 (13)	0.0308 (3)	
N1	0.40867 (8)	0.38809 (9)	0.80434 (14)	0.0254 (3)	
C1	0.53672 (9)	0.37846 (11)	0.90859 (17)	0.0251 (3)	
C2	0.58415 (10)	0.36524 (11)	1.03887 (19)	0.0281 (4)	
H2	0.6393 (11)	0.3633 (14)	1.024 (2)	0.034 (5)*	
C3	0.55465 (10)	0.35284 (12)	1.18543 (19)	0.0303 (4)	
H3	0.5872 (10)	0.3427 (14)	1.279 (2)	0.039 (5)*	
C4	0.47679 (10)	0.35250 (12)	1.20670 (18)	0.0298 (4)	
H4	0.4558 (10)	0.3457 (14)	1.309 (2)	0.032 (5)*	
C5	0.42866 (10)	0.36311 (12)	1.08128 (18)	0.0277 (4)	
H5	0.3734 (10)	0.3620 (13)	1.098 (2)	0.031 (5)*	
C6	0.45761 (9)	0.37626 (11)	0.93073 (17)	0.0246 (3)	
C7	0.43350 (10)	0.39750 (11)	0.65289 (17)	0.0270 (3)	
C8	0.51480 (10)	0.40045 (11)	0.63199 (18)	0.0269 (3)	
H8	0.5319 (10)	0.4116 (14)	0.529 (2)	0.036 (5)*	
C9	0.56382 (9)	0.39364 (11)	0.75165 (17)	0.0260 (3)	
C10	0.32666 (10)	0.39452 (12)	0.83043 (19)	0.0285 (4)	
H10A	0.3196 (10)	0.4370 (13)	0.927 (2)	0.029 (4)*	
H10B	0.3059 (10)	0.4312 (14)	0.736 (2)	0.033 (5)*	
C11	0.28830 (10)	0.29203 (12)	0.8470 (2)	0.0295 (4)	
H11A	0.3155 (10)	0.2489 (14)	0.924 (2)	0.027 (4)*	
H11B	0.2890 (10)	0.2551 (14)	0.742 (2)	0.035 (5)*	
C12	0.20666 (10)	0.30564 (14)	0.8979 (2)	0.0351 (4)	
H12A	0.2056 (11)	0.3463 (15)	1.001 (2)	0.046 (6)*	
H12B	0.1785 (11)	0.3465 (16)	0.818 (2)	0.046 (6)*	
C13	0.16396 (11)	0.20777 (15)	0.9228 (2)	0.0390 (4)	
H13A	0.1920 (11)	0.1658 (16)	1.009 (2)	0.049 (6)*	
H13B	0.1668 (11)	0.1653 (16)	0.826 (2)	0.045 (6)*	
C14	0.08281 (12)	0.22432 (18)	0.9724 (3)	0.0485 (5)	
H14A	0.0815 (13)	0.2635 (19)	1.075 (3)	0.070 (7)*	
H14B	0.0564 (13)	0.2674 (19)	0.896 (3)	0.068 (7)*	
C15	0.03901 (15)	0.1279 (2)	0.9986 (4)	0.0656 (7)	
H15A	0.0625 (16)	0.085 (2)	1.085 (3)	0.086 (9)*	
H15B	−0.0159 (16)	0.1416 (19)	1.031 (3)	0.076 (8)*	
H15C	0.0378 (15)	0.089 (2)	0.895 (3)	0.086 (9)*	
C16	0.64634 (10)	0.40936 (12)	0.71935 (18)	0.0280 (4)	
C17	0.74124 (10)	0.40261 (14)	0.5285 (2)	0.0329 (4)	
H17A	0.7783 (11)	0.3728 (14)	0.602 (2)	0.036 (5)*	
H17B	0.7456 (11)	0.4763 (17)	0.533 (2)	0.044 (6)*	
C18	0.75172 (10)	0.36435 (13)	0.3648 (2)	0.0325 (4)	
H18A	0.7468 (11)	0.2897 (16)	0.365 (2)	0.046 (6)*	
H18B	0.7102 (11)	0.3912 (14)	0.299 (2)	0.037 (5)*	
C19	0.82624 (11)	0.39951 (15)	0.2953 (2)	0.0352 (4)	
H19A	0.8683 (12)	0.3685 (16)	0.350 (2)	0.050 (6)*	
H19B	0.8308 (11)	0.4748 (17)	0.311 (2)	0.049 (6)*	
C20	0.83340 (11)	0.37729 (15)	0.1223 (2)	0.0385 (4)	
H20A	0.8297 (12)	0.3045 (18)	0.106 (2)	0.052 (6)*	
H20B	0.7894 (12)	0.4082 (16)	0.068 (2)	0.047 (6)*	
C21	0.90568 (12)	0.41679 (18)	0.0503 (2)	0.0440 (5)	
H21A	0.9504 (13)	0.3819 (17)	0.102 (3)	0.059 (7)*	
H21B	0.9100 (14)	0.490 (2)	0.075 (3)	0.069 (7)*	
C22	0.90816 (15)	0.4025 (2)	−0.1249 (3)	0.0579 (6)	
H22A	0.9029 (15)	0.332 (2)	−0.154 (3)	0.080 (9)*	
H22B	0.8651 (16)	0.439 (2)	−0.178 (3)	0.077 (8)*	
H22C	0.9566 (14)	0.4306 (19)	−0.170 (3)	0.069 (7)*	

### Atomic displacement parameters (Å^2^)

**Table:**

	*U*^11^	*U*^22^	*U*^33^	*U*^12^	*U*^13^	*U*^23^
O1	0.0371 (7)	0.0385 (7)	0.0258 (6)	−0.0021 (5)	−0.0026 (5)	0.0022 (5)
O2	0.0351 (7)	0.0463 (7)	0.0337 (6)	−0.0085 (6)	0.0025 (5)	−0.0066 (5)
O3	0.0322 (7)	0.0304 (6)	0.0301 (6)	−0.0038 (5)	0.0069 (5)	−0.0041 (5)
N1	0.0296 (8)	0.0219 (6)	0.0248 (7)	−0.0008 (5)	0.0015 (5)	−0.0004 (5)
C1	0.0335 (9)	0.0164 (7)	0.0253 (8)	−0.0021 (6)	0.0003 (6)	−0.0015 (5)
C2	0.0324 (10)	0.0213 (7)	0.0307 (8)	−0.0010 (7)	−0.0018 (7)	−0.0003 (6)
C3	0.0402 (10)	0.0247 (8)	0.0260 (8)	−0.0032 (7)	−0.0036 (7)	0.0005 (6)
C4	0.0443 (11)	0.0228 (8)	0.0223 (8)	−0.0037 (7)	0.0036 (7)	−0.0014 (6)
C5	0.0352 (10)	0.0221 (7)	0.0259 (8)	−0.0025 (7)	0.0037 (7)	−0.0018 (6)
C6	0.0344 (9)	0.0166 (7)	0.0230 (7)	−0.0006 (6)	0.0006 (6)	−0.0009 (5)
C7	0.0363 (10)	0.0198 (7)	0.0249 (8)	−0.0010 (6)	0.0004 (7)	0.0009 (6)
C8	0.0346 (9)	0.0223 (8)	0.0240 (8)	−0.0023 (6)	0.0044 (7)	0.0001 (6)
C9	0.0336 (9)	0.0177 (7)	0.0267 (8)	−0.0012 (6)	0.0033 (7)	−0.0027 (6)
C10	0.0293 (9)	0.0266 (8)	0.0297 (8)	0.0024 (7)	0.0027 (7)	0.0014 (7)
C11	0.0302 (9)	0.0276 (8)	0.0308 (8)	−0.0008 (7)	0.0016 (7)	0.0002 (7)
C12	0.0316 (10)	0.0358 (9)	0.0380 (10)	0.0001 (7)	0.0033 (8)	0.0040 (8)
C13	0.0340 (10)	0.0405 (10)	0.0426 (10)	−0.0039 (8)	0.0017 (8)	0.0051 (8)
C14	0.0342 (11)	0.0584 (13)	0.0530 (13)	−0.0058 (10)	0.0038 (10)	0.0092 (10)
C15	0.0428 (14)	0.0811 (18)	0.0730 (18)	−0.0203 (13)	0.0015 (13)	0.0178 (15)
C16	0.0335 (9)	0.0224 (7)	0.0282 (8)	−0.0006 (7)	0.0025 (7)	−0.0001 (6)
C17	0.0296 (10)	0.0343 (10)	0.0347 (9)	−0.0024 (7)	0.0053 (7)	−0.0017 (7)
C18	0.0326 (10)	0.0319 (9)	0.0332 (9)	−0.0029 (7)	0.0027 (8)	−0.0019 (7)
C19	0.0304 (10)	0.0415 (10)	0.0336 (9)	−0.0011 (8)	0.0037 (8)	−0.0016 (7)
C20	0.0372 (11)	0.0432 (11)	0.0350 (10)	−0.0033 (8)	0.0038 (8)	−0.0031 (8)
C21	0.0374 (12)	0.0568 (13)	0.0379 (10)	−0.0043 (10)	0.0080 (9)	−0.0041 (9)
C22	0.0516 (15)	0.0812 (18)	0.0410 (12)	−0.0106 (13)	0.0126 (11)	−0.0032 (12)

### Geometric parameters (Å, º)

**Table:**

O1—C7	1.2388 (19)	C12—H12A	1.04 (2)
O2—C16	1.2096 (19)	C12—H12B	1.01 (2)
O3—C16	1.3376 (19)	C13—C14	1.515 (3)
O3—C17	1.451 (2)	C13—H13A	1.05 (2)
N1—C7	1.380 (2)	C13—H13B	1.00 (2)
N1—C6	1.395 (2)	C14—C15	1.511 (3)
N1—C10	1.471 (2)	C14—H14A	1.02 (3)
C1—C2	1.408 (2)	C14—H14B	0.98 (3)
C1—C6	1.413 (2)	C15—H15A	1.02 (3)
C1—C9	1.447 (2)	C15—H15B	1.03 (3)
C2—C3	1.374 (2)	C15—H15C	1.02 (3)
C2—H2	0.985 (19)	C17—C18	1.507 (2)
C3—C4	1.390 (2)	C17—H17A	0.992 (19)
C3—H3	0.998 (19)	C17—H17B	0.98 (2)
C4—C5	1.378 (2)	C18—C19	1.522 (2)
C4—H4	0.956 (18)	C18—H18A	0.99 (2)
C5—C6	1.404 (2)	C18—H18B	0.99 (2)
C5—H5	0.988 (18)	C19—C20	1.521 (2)
C7—C8	1.451 (2)	C19—H19A	0.97 (2)
C8—C9	1.346 (2)	C19—H19B	1.01 (2)
C8—H8	0.945 (19)	C20—C21	1.515 (3)
C9—C16	1.502 (2)	C20—H20A	0.98 (2)
C10—C11	1.525 (2)	C20—H20B	0.99 (2)
C10—H10A	1.011 (18)	C21—C22	1.518 (3)
C10—H10B	1.015 (18)	C21—H21A	1.02 (2)
C11—C12	1.521 (2)	C21—H21B	1.00 (3)
C11—H11A	0.997 (17)	C22—H22A	0.97 (3)
C11—H11B	1.022 (18)	C22—H22B	1.01 (3)
C12—C13	1.516 (3)	C22—H22C	1.01 (3)
			
C16—O3—C17	115.00 (13)	C12—C13—H13B	109.7 (12)
C7—N1—C6	123.04 (14)	H13A—C13—H13B	105.0 (16)
C7—N1—C10	117.09 (13)	C15—C14—C13	114.0 (2)
C6—N1—C10	119.84 (13)	C15—C14—H14A	106.8 (14)
C2—C1—C6	118.59 (14)	C13—C14—H14A	109.8 (14)
C2—C1—C9	124.05 (15)	C15—C14—H14B	110.3 (14)
C6—C1—C9	117.37 (14)	C13—C14—H14B	110.2 (14)
C3—C2—C1	121.07 (16)	H14A—C14—H14B	105 (2)
C3—C2—H2	119.7 (11)	C14—C15—H15A	111.5 (16)
C1—C2—H2	119.3 (11)	C14—C15—H15B	112.2 (15)
C2—C3—C4	120.01 (16)	H15A—C15—H15B	106 (2)
C2—C3—H3	122.4 (11)	C14—C15—H15C	107.7 (16)
C4—C3—H3	117.5 (11)	H15A—C15—H15C	111 (2)
C5—C4—C3	120.45 (16)	H15B—C15—H15C	108 (2)
C5—C4—H4	118.9 (11)	O2—C16—O3	123.42 (15)
C3—C4—H4	120.6 (11)	O2—C16—C9	124.59 (15)
C4—C5—C6	120.45 (16)	O3—C16—C9	111.96 (13)
C4—C5—H5	119.7 (10)	O3—C17—C18	108.01 (14)
C6—C5—H5	119.9 (10)	O3—C17—H17A	108.2 (11)
N1—C6—C5	120.25 (15)	C18—C17—H17A	112.2 (11)
N1—C6—C1	120.34 (14)	O3—C17—H17B	108.5 (12)
C5—C6—C1	119.41 (14)	C18—C17—H17B	111.1 (11)
O1—C7—N1	121.22 (15)	H17A—C17—H17B	108.8 (16)
O1—C7—C8	122.79 (15)	C17—C18—C19	111.86 (15)
N1—C7—C8	115.96 (14)	C17—C18—H18A	108.8 (11)
C9—C8—C7	122.71 (15)	C19—C18—H18A	112.4 (12)
C9—C8—H8	121.0 (11)	C17—C18—H18B	108.6 (11)
C7—C8—H8	116.2 (11)	C19—C18—H18B	107.9 (11)
C8—C9—C1	120.44 (15)	H18A—C18—H18B	107.1 (15)
C8—C9—C16	118.29 (14)	C20—C19—C18	113.51 (15)
C1—C9—C16	121.13 (14)	C20—C19—H19A	109.0 (12)
N1—C10—C11	113.70 (13)	C18—C19—H19A	110.2 (12)
N1—C10—H10A	106.4 (10)	C20—C19—H19B	108.3 (11)
C11—C10—H10A	111.3 (10)	C18—C19—H19B	108.6 (12)
N1—C10—H10B	105.1 (10)	H19A—C19—H19B	107.0 (17)
C11—C10—H10B	110.0 (10)	C21—C20—C19	113.87 (16)
H10A—C10—H10B	110.2 (14)	C21—C20—H20A	109.8 (13)
C12—C11—C10	110.15 (14)	C19—C20—H20A	109.0 (12)
C12—C11—H11A	109.5 (10)	C21—C20—H20B	109.1 (12)
C10—C11—H11A	111.0 (10)	C19—C20—H20B	108.0 (12)
C12—C11—H11B	108.9 (10)	H20A—C20—H20B	106.7 (17)
C10—C11—H11B	109.7 (11)	C20—C21—C22	112.87 (18)
H11A—C11—H11B	107.6 (14)	C20—C21—H21A	108.7 (13)
C13—C12—C11	114.40 (15)	C22—C21—H21A	110.7 (13)
C13—C12—H12A	108.3 (11)	C20—C21—H21B	108.2 (14)
C11—C12—H12A	109.1 (11)	C22—C21—H21B	109.4 (14)
C13—C12—H12B	108.1 (12)	H21A—C21—H21B	106.7 (19)
C11—C12—H12B	109.7 (12)	C21—C22—H22A	112.0 (16)
H12A—C12—H12B	107.0 (16)	C21—C22—H22B	111.1 (15)
C14—C13—C12	112.89 (17)	H22A—C22—H22B	106 (2)
C14—C13—H13A	109.1 (11)	C21—C22—H22C	111.1 (14)
C12—C13—H13A	108.5 (11)	H22A—C22—H22C	110 (2)
C14—C13—H13B	111.3 (12)	H22B—C22—H22C	107 (2)
			
C6—C1—C2—C3	−1.6 (2)	C7—C8—C9—C16	−173.12 (13)
C9—C1—C2—C3	178.89 (14)	C2—C1—C9—C8	176.52 (15)
C1—C2—C3—C4	0.5 (2)	C6—C1—C9—C8	−3.0 (2)
C2—C3—C4—C5	0.9 (2)	C2—C1—C9—C16	−7.8 (2)
C3—C4—C5—C6	−1.2 (2)	C6—C1—C9—C16	172.65 (13)
C7—N1—C6—C5	−177.49 (14)	C7—N1—C10—C11	99.07 (16)
C10—N1—C6—C5	4.7 (2)	C6—N1—C10—C11	−82.96 (17)
C7—N1—C6—C1	3.2 (2)	N1—C10—C11—C12	171.38 (14)
C10—N1—C6—C1	−174.68 (13)	C10—C11—C12—C13	−178.17 (15)
C4—C5—C6—N1	−179.30 (14)	C11—C12—C13—C14	−179.66 (16)
C4—C5—C6—C1	0.1 (2)	C12—C13—C14—C15	−179.8 (2)
C2—C1—C6—N1	−179.37 (13)	C17—O3—C16—O2	−8.3 (2)
C9—C1—C6—N1	0.2 (2)	C17—O3—C16—C9	169.68 (13)
C2—C1—C6—C5	1.3 (2)	C8—C9—C16—O2	143.61 (17)
C9—C1—C6—C5	−179.14 (13)	C1—C9—C16—O2	−32.2 (2)
C6—N1—C7—O1	178.45 (14)	C8—C9—C16—O3	−34.38 (19)
C10—N1—C7—O1	−3.6 (2)	C1—C9—C16—O3	149.83 (14)
C6—N1—C7—C8	−3.5 (2)	C16—O3—C17—C18	−175.71 (14)
C10—N1—C7—C8	174.39 (13)	O3—C17—C18—C19	174.17 (14)
O1—C7—C8—C9	178.56 (15)	C17—C18—C19—C20	−170.54 (17)
N1—C7—C8—C9	0.6 (2)	C18—C19—C20—C21	177.03 (17)
C7—C8—C9—C1	2.7 (2)	C19—C20—C21—C22	−174.6 (2)

### Hydrogen-bond geometry (Å, º)

**Table:**

*D*—H···*A*	*D*—H	H···*A*	*D*···*A*	*D*—H···*A*
C4—H4···O1^i^	0.960 (17)	2.475 (17)	3.3670 (19)	154.7 (15)

Symmetry code: (i) *x*, *y*, *z*+1.
